# Exploring the Influencing Factors of the Recreational Utilization and Evaluation of Urban Ecological Protection Green Belts for Urban Renewal: A Case Study in Shanghai

**DOI:** 10.3390/ijerph181910244

**Published:** 2021-09-29

**Authors:** Weiqi Zhao, Yun Wang, Dan Chen, Ling Wang, Xiaomin Tang

**Affiliations:** School of Design, Shanghai Jiao Tong University, Shanghai 200240, China; by19970519@sjtu.edu.cn (W.Z.); wwlling@sjtu.edu.cn (L.W.); xmtang@sjtu.edu.cn (X.T.)

**Keywords:** green belt, recreational utilization, potential evaluation, social media data, multivariate regression model, shanghai

## Abstract

With the continuous expansion of urban construction land, the green belts aiming for ecological protection have ensured a sustainable and effective function of regional ecosystem services. At the same time, these ecological green belts are expected to develop their compound service potentials with the development of cities. In order to meet the increasing demand of urban residents for the recreational utilization of urban green space, the primary function of the ecological green belts has transformed from being purely ecological to a combination of being ecological and recreational. Based on social media data, which has the characteristics of a large amount of accessible geographic information, this study used multiple regression models to analyze the recreational utilization intensity of ecological protection green belts with a case study in the green belt of Shanghai, China. The research results showed that the internal elements (total external area, water area, etc.) of the Shanghai green belt have positive correlations with its recreational utilization. The impact of external factors was inconclusive on the recreational utilization of the outer forest belt (the number of subway stations in accessibility factors was negatively correlated; the number of cultural facilities and the number of restaurants in the surrounding service facilities were positively related). Combined with the “Shanghai City Master Plan (2017–2035)”, this study suggests potential zones for the recreational transformation of the Shanghai green belt, provides a theoretical and practical basis for improving the recreational utilization of an urban ecological protection green belt and contributes to the sustainable development of ecological protection green belts in high-density cities.

## 1. Introduction

Regional ecosystem services guarantee the health and well-being of urban and rural residents. The urban green space system, comprised of large ecological patches, landscape ecological corridors and city parks, is the foundation to ensure the continuous performance of services, and the ecological protection green belt, as a style of ecological corridor, is a key component. Boston’s well-known Emerald Necklace has evolved from the initial protection of natural ecology and connection of city parks to a recreational greenway with ecological significance [1]. The idea of an ecological protection green belt is usually considered to have originated from Ebenezer Howard’s development of a “garden city” around London in the early 20th century [2,3,4]. Howard’s initial strategy was to curb urban sprawl. This concept was first implemented in the Greater London Plan in 1945. This plan was a great success, and the concept of the green belt was thus widely imitated in the UK and elsewhere afterwards [5,6]. The appearance of the ecological protection green belt is used to effectively control the growth of urban land and serve ecological functions, including maintaining bio-diversity, improving overall protection and utilization of environmental resources in marginal areas [7], regulating the urban microclimate and absorbing pollutants [8]. In recent years, researchers have paid more attention to the compound functions of a green belt around a city, and they have studied new planning policies for green belts to ensure the sustainable development of an ecological protection green belt to contribute to current cities [9].

With the increasing requirements for healthier lifestyles, green recreational space has become essential for urban residents [10]. More and more studies have shown that entering the green space plays an important role in people’s health and well-being [11,12]. In addition to their widely studied role in urban microclimate and the absorption of pollutants [8], urban green spaces provide urban residents with a place for sports and recreation and improve their physical and mental health [11,13]. People’s recreational requirements for urban green spaces are increasing with improved living levels [14,15,16,17]. However, to accommodate growing populations, governments have continued to expand urban land at the expense of green recreational space [18,19,20]. The conflict between the decrease in recreational space and the increase in citizens’ demand for recreation has intensified [21]. The existence of urban protective green belts is one of the largest green space resources in a city. The planning of urban green belts in Hong Kong and other places proves that ecological protective green belts have a certain degree of flexibility and that they can play different roles in different spaces throughout a city, which shows changeability in their planning principles [4]. The recreational utilization function is also an important planning purpose of a green belt. It is essential for an urban ecological protection green belt to transform in response to the changes in a modern city. The recreational utilization function of the urban protective green belt will make a significant contribution to balancing the negative impacts of rapid urbanization on the health and well-being of residents.

The research on the factors affecting the recreational use of the green belts has a strong guiding significance for the planning of recreational transformation. Many researchers have studied the factors affecting the recreational utilization of green space. However, there is no consensus on the influencing factors. Many studies have focused on polygonal green space, which can also be applied to striped green space, and areas [22], attractiveness [23,24,25], accessibility [26,27] and the amount of green space [28] have positive impacts on polygonal green space. In the study by Zhang et al., the attractiveness of area size for residents to enter the park was second only to the entrance fee, and this result explained 57% of the survey data in their research; another 27% of the survey data showed that the inconvenience of transportation would lead to a decline in the utilization rate of the park, and at this time the positive impact of factors such as park area and cost would not be obvious [29]. Hooper’s research results showed that most people will use parks and green spaces within 1500 m of their homes that are easily accessible, which showed that accessibility is an important influencing factor [30]. Donahue found that water features are closely related to the utilization of parks through analyzing Twitter photos [31]. These previous studies indicate that residents’ recreational utilization of urban parks is inextricably linked to the area, landscape, accessibility and the surrounding environment. Large-scale natural parks full of natural scenery are very popular with residents; at the same time, community parks that are easy to reach and have more exquisite designs are also very important for the entertainment and leisure activities of nearby residents [32,33].

Reviewing the literature, most research on urban green space relies on traditional survey methods, such as a questionnaire survey of random or representative sample users to measure the recreational use of urban green space and to explore the factors affecting recreational use [24,31,33,34,35]. However, such methods are usually limited by several factors, including the time selection of the investigation, the ability to interpret questions and the number of samples. For a large research area, material and human resources are expensive [36]. More importantly, in the context of the coronavirus (COVID-19) pandemic, face-to-face investigations are not feasible under the current situation. In the past few years, the number of studies using rich geographic information resources provided by social media to evaluate the recreational use of green park space has surged [37,38,39]. Social media data can aid the analysis of user attributes to study the relationship between people and the environment in urban parks [40]. Social media data can also be used to measure the utilization of urban green space and evaluate its influential factors [14,18,31,41]. Inspired by the demand for large amounts of green space usage data with a long time span and the research methods mentioned above, we decided to estimate the recreational use of urban green space through social media data in this study.

On the basis of protecting the existing ecological pattern of the ecological protection green belt, how shall we improve the recreational service function of the green belt in a targeted manner to meet the residents’ increasing demand for recreational green space? The main purpose of this article is to explore the factors that affect recreational utilization of the green belt and contribute to the planning of the recreational transformation to contribute to the sustainable development of ecological protection green belts in the context of urban renewal. Previous studies have discussed the factors affecting the recreational utilization of urban green space. In this study, we assumed that these influencing factors could also be used for the study of the recreational utilization of protective green belts. Therefore, it was assumed that the internal attributes, accessibility, and surrounding service facilities of ecological protection green belts would affect residents’ recreational utilization, which also determined the potential location of an urban green belt recreation transformation. The Shanghai green belt is an ecological protection green belt that was built in 1994 to prevent the unlimited expansion of the city. In this work, the Shanghai green belt was used as part of an empirical study to explore the factors that actually affected the recreational utilization of the ecological protection green belt, and propose planning guidance for the transformation of recreational utilization in the city to explore the sustainable development of the green belt in response to the changes in the recreational demand of today’s residents and to improve the sustainable development of residents’ health.

## 2. Methodology

In order to explore the factors affecting the recreational utilization of the Shanghai green belt, a multiple regression model was used in this study. Sina Weibo sign-in data was used to quantify the recreational utilization of the green belt (dependent variable) and the impact factor (independent variable) in the regression model.

### 2.1. Study Area

The Shanghai government approved the construction of the green belt in 1994. It is a vital place for entertainment, and is also an open green belt that provides ecological protection and isolates urban and rural development. The construction of the green belt effectively prevented the unlimited expansion of the city and brought significant environmental and social benefits. It also has significant ecological and economic benefits, such as adjusting temperature and humidity (e.g., alleviating the urban heat island effect) and purifying the atmospheric environment [21,42,43]. With the expansion of construction land in the central urban area, the location of the green belt was no longer in the boundary area. The acceleration of urbanization and the development of the urban economy also led to population increase in the urban area. The urban residential areas have been concentrated within the outer ring, and gradually developed near the green belt, especially in the western part. After 2005, residential areas with green spaces began to be built around the green belt. After twenty-five years of rapid urban growth, restraining urban expansion is no longer the primary function of the green belt. The green belt is no longer able to meet the needs of citizens for its purely ecological purposes [21,44].

**Figure 1:**
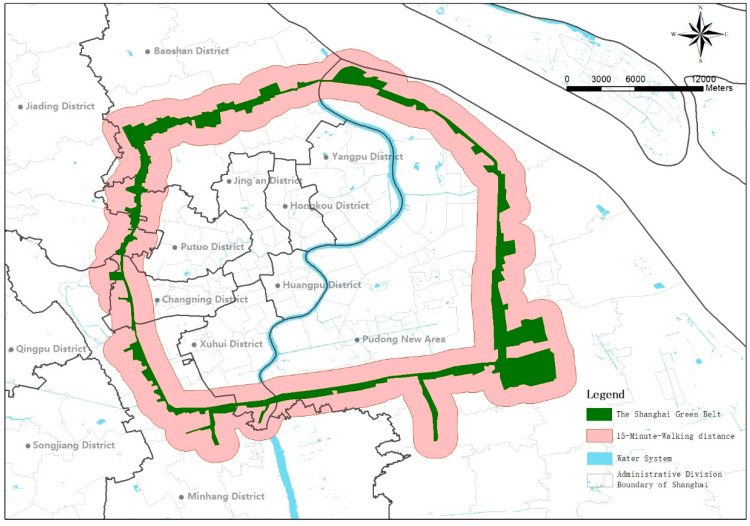
The green belt and the 15-min walking distance buffers (about 1500 m) on both sides of the green belt.

The Shanghai green belt is 98 kilom long and has a width of at least 500 m. The green belt passes through seven administrative regions including Baoshan, Minhang and Changning District, with a total area of about 72.41 km^2^. The green space can be classified into two types. The first type is a 500-m-wide shelter forest model, including a 100-m-wide open, pure forest belt dominated by trees and a 400-m-wide green belt built along the road’s outer area. The landscape of the Shanghai green belt is weak, lacking infrastructure. The second type mainly includes botanical gardens, forest parks, green entertainment parks and other parks built on the green belt. These parks make up for the lack of green open space and serve as community parks for the surrounding residents (Figure 1, The Shanghai green belt).

Given the green belt’s overall service facilities and aesthetic level, the green space is not enough to attract citizens from the city for recreational activities. In the preliminary investigation, the target population had to be mainly surrounding residents. According to the “Shanghai 15-min walk living circle” policy, in order to achieve the goal of constructing a multi-type, multi-level, walkable and public urban network space by the year of 2035, the green belt is responsible for providing green open space to dense residential areas on both sides—a vital function of available green space within 15 min. A 15-min walking distance is taken as the acceptable service range of the green belt based on residents’ daily recreational needs and behaviors. Therefore, in this study, the green belt and the 15-min walking distances (about 1500 m) on both sides were examined (Figure 1, 15-min walking distance).

### 2.2. Data Collection

#### 2.2.1. Social Media Data

The recreational use data in this study came from the Sina Weibo sign-in data from December 2011 to July 2020 (Table 1). Sina Weibo, usually regarded as Twitter in China, is one of the most important social media platforms and one of the largest social media platforms in China [45]. In 2020, the average number of daily active users of Weibo was 229 million, and the number of monthly active users was 523 million. Weibo is in the top tier of China’s social media platforms and provides a rich database for research material, covering the range of residents’ activities in all four seasons. We signed an agreement with Sina Weibo to purchase data, provided by Application Programming Interface (API), and obtained more than 3 million pieces of Weibo sign-in data from its establishment from December 2011 to July 2020. Sina Weibo records the location and statuses of users. Each record of the sign-in data includes the following fields:

- User ID: identity code of anonymous user;
- Longitude: longitude of the user’s activity point;
- Latitude: latitude of the user’s activity point;
- Time: time of the user’s activity.

#### 2.2.2. Basic Data Related to the Green Belt

Other basic data related to the green belt (Table 2) mainly includes information on Shanghai’s urban road network, bus stations, subway stations, urban water systems and points of interest (POI) near the green belt. According to the Shanghai Statistical Yearbook [46], Shanghai’s road network and public transportation system have undergone minor changes from 2011 to 2020 [47]. The increase in roads is within 10% and mainly in suburban roads, and the impact on the periphery of the green belt is relatively small. The data on bus stops, subway stations and POI points will match the Weibo check-in data, so the latest basic data that can be obtained is selected. In order to obtain the geographic information data for the city, the area data for Shanghai was extracted from OpenStreetMap [48]. The POI data came from the Weibo application, and the data could be used to reflect the business prosperity around the green belt.

### 2.3. Definition and Evaluation Method for the Recreational Utilization of the Green Belt

Recreational utilization, which is an essential function of urban green space [49,50], relies on natural and human resources as well as other attractions as carriers to provide residents with the function of achieving physical and mental rest within a certain travel time or distance [29,51,52]. In previous studies, researchers have proven that the density of social media posts is positively correlated with the number of actual visits [53], and there are empirical studies that have used Weibo check-in data to quantify the number of visits to urban parks and the recreational preferences of residents [29,54]. For this study, it was believed that the user check-in data in the green belt represented the residents’ visits and utilization, which could be used to define the recreational utilization. Therefore, the Weibo check-in data for residents in the green belt was used as a quantitative indicator to measure the recreational utilization of the green belt.

Through this research, multiple regression equations affecting the recreational utilization of the green belt were established. The results of the equations were combined with related urban planning strategies, and the future leisure use of the urban green belt was calculated by incorporating the values of related independent variables into the regression equation in order to propose the recreational evaluation of the green belt.

### 2.4. Determination and Calculation of Impact Factors

Through a literature review and investigation, it was found that factors affecting the green belt around the city included internal factors, accessibility and surrounding service facilities. The internal influencing factors were attributes related to the initial design of the green belt, including the size of the green area, the size of the water body, and the degree of beauty, which was determined by scoring the beauty of current photos. Accessibility is a measure connecting the supply and demand for recreational services. Convenient transportation will increase the frequencies and opportunities of contact between residents and green spaces. It can be analyzed from the area of the road network, the number of bus stations, and the number of subway stations [55]. Surrounding service facilities refer to the influencing factors located outside a green belt, which can be analyzed for residential areas, shopping spots, restaurants, cultural facilities, tourist spots, and entertainment facilities. In this study, we used the thirteen impact factors shown in Table 3.

Other basic data related to the green belt (Table 2) was used to calculate internal factors and accessibility. Through the ArcGIS platform, using the shape of the green belt and the water area, the area of the required range and the number of roads, bus stops and subway stations within the scope were calculated. The degree of beauty was calculated by the Scenic Beauty Estimation Method (SBE method), which is often used to evaluate landscapes aesthetically [56,57]. The selection of sample plots was carried out based on the principles of full coverage in different administrative divisions, time of establishment, diversity of plant community types, and richness of plant species. After ensuring that the sample plots cover all the administrative regions, the number of sample plots were distributed proportionally according to the length of the green belt belonging to each administrative region. Finally, 101 sample plots of the green belt were selected to represent different sections. The public’s perception of the beauty of the green belt was measured by taking photos and evaluating photos by professionals and non-professionals. The degree of beauty reflected the current visual situation of the green belt [58]. The combination of social media data (Table 1) and the basic data related to the green belt (Table 2) was used to determine the surrounding service facilities. A summary of the statistics of the variables used in this study is shown in Table 4.

### 2.5. Multiple Regression Model

In order to explore the relationship between the influencing factors and the recreational utilization of the green belt, a multiple regression model of the recreational utilization was established. In this model, the dependent variable of the park recreation utilization was estimated using Weibo check-in data, and the independent variables (influencing factors) of the green belt recreation utilization were determined using the thirteen attributes of the green belt. It should be noted that seasonal changes brought changes in the plant seasons and temperature. Therefore, the four seasons of spring, summer, autumn and winter were introduced as control variables to illustrate the potential seasonal effects of the recreational use of the green belt. According to Shanghai’s climate changes, spring was defined from March to May, summer was defined from June to August, autumn was defined from September to November, and winter was defined from December to February. Based on the above variables, a logarithmic multiple regression model (1) was used:

(1) $$ln\left(Y\right)=b_{0}+b_{1}ln\left(X_{1}\right)+b_{2}ln\left(X_{2}\right)+\cdots +b_{13}ln\left(X_{13}\right)$$

where *Y* is the number of Weibo check-in data points, *Xi* (*i* = 1, 2…, 13) is the influencing factor and *bi* (*i* = 0, 1,2…, 13) is the coefficient to be solved. ln stands for log transformation. This is a special data transformation method, which can reduce the absolute value of the data to facilitate calculation. In this study, the absolute value of the area data and the sign-in data are both large, while the absolute value of the service facility data is small, and the magnitude of the independent variable is inconsistent. Therefore, the logarithmic transformation of the data is carried out to eliminate this large difference in magnitude. The study calculated both the logarithmic model and the dual logarithmic model and selected the dual logarithmic model with a higher model fitting result. The entirety of the statistical analysis was performed using IBM SPSS Statistics 24 (SPSS Inc., Chicago, IL, USA).

Multiple regression models and logarithmic regression models have been widely used in previous studies to detect the statistical relationships between the utilization of green space and the impact factors [59,60]. In this study, four multiple regression models (the linear form, semi-logarithmic form, logarithmic linear form and logarithmic form of the multiple regression equation) were preliminarily tested during model selection. The logarithmic model was the most appropriate since it had the highest degree of fit and explanation.

## 3. Results

### 3.1. The Relationship between Recreational Utilization and the Influencing Factors of the Green Belt

According to the selection results of the above models, a logarithmic model was adopted and the natural logarithm of the number of check-ins and the natural logarithm of all numerical variables were used in a stepwise regression. The model was analyzed and tested on the judgment coefficient, the probability value of the F statistic, the variance expansion index VIF value, the homogeneity of the variance and the residual value. The test results showed that the built model had a reasonable degree of fit and high explanatory power. The fitting of the sample data was statistically significant, and it could be used to analyze and explain the impact of various variables on the recreational utilization of the Shanghai green belt.

The results of the regression model (Table 5) showed that the number of subway stations passed the 5% significance level test, while the water area, 1500 m service area, number of residential areas, number of restaurants and number of cultural facilities all passed the 1% significance level test. Among these six factors, the number of residential areas and the number of subway stations had significantly negative impacts on the recreational utilization of the green belt, while the number of cultural facilities, the areas of the water bodies, the number of restaurants and the area had significantly positive effects.

The Shanghai green belt is divided into two parts: the pure forest belt and the park built on the green belt. Therefore, there was likely to be a difference between the recreational utilization of the park and the pure forest belt. Therefore, 188 groups of samples were selected, with 56 groups of samples from the parks and 132 groups from the pure forest belt. Then, a regression model analysis was performed on each of these two groups of data (Table 6). In addition to the difference in the internal impact factors, the results of the built park and the pure forest belt were also different in terms of accessibility. The results of the built park showed that for every 1% increase in road area, recreational utilization will increase by 21.227%; for every 1% increase in the number of bus stops, recreational utilization will increase by 11.587%. For the results of the pure forest belts, the road area and the number of bus stops were not significant.

In addition, the policy of “Guidelines for Environmental Landscape Design of Residential Areas (Trial Draft)” was believed to be the cause for why Shanghai’s real estate market was in an overheating stage in 2005 [61]. Since then, the supporting facilities of commercial housing have been rapidly improved, the green landscape of residential areas has been paid attention to and the improvement of the environment may have had a certain impact on the results of the recreational utilization of the green belt. Therefore, the third regression analyzed the impact of the construction year of the residential area around the green belt on the recreational utilization (Table 7). In this model, the difference between pure forest belts and parks around the city was not considered because of the aim of obtaining the impact of the construction year of the residential area on the recreational utilization. The residential buildings were divided into those built before and after 2005, and the model analysis was performed again. The results of the model showed that for residential areas built after 2005, every 1% increase in the number of residential areas will lead to a 0.681% drop in recreational use. In the model before 2005, residential areas did not have a significant impact. 

### 3.2. Recreational Potential Evaluation

**Figure 2:**
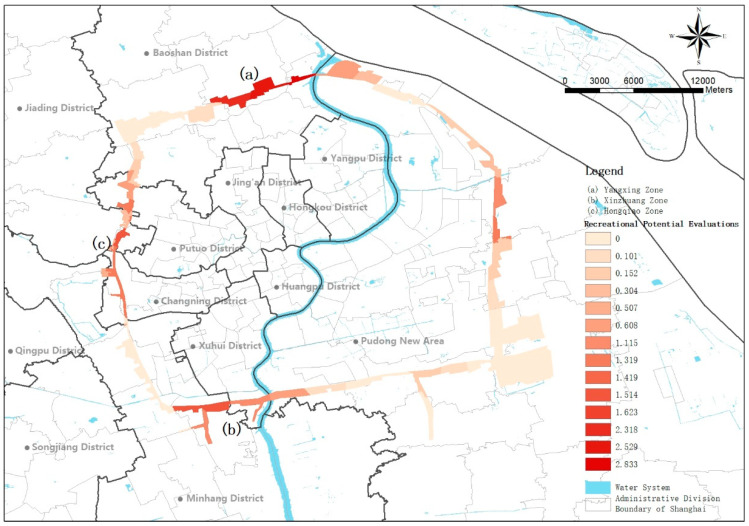
Recreational potential evaluations of recreation utilization along the Shanghai green belt.

According to the results above, factors such as residential areas, cultural facilities and accessibility significantly affect the recreational use of the green belt. Combined with the land use plan given in the “Shanghai City Master Plan (2017–2035)” and the strategic guidance maps of each administrative region, the recreational potential of the green belt was calculated. The recreational potential evaluations of the recreation utilization along the Shanghai green belt are shown in Figure 2. The Yanghang zone (a), Xinzhuang zone (b) and Hongqiao zone (c) were the points with the most recreational potential in the green belt. On one hand, in the “Shanghai City Master Plan (2017–2035)”, the Hongqiao hub at the junction of Minhang and Changning District near the green belt would become an international (national) transportation hub, while the Yanghang and Xinzhuang hubs in Baoshan and Minhang District were planned to be a city-level transportation hub. Combining the strategic guidance maps of various administrative regions, it was found that a large number of residential areas will be formed near these three transportation hubs, as well as cultural facilities and other public service facilities. On the other hand, high-quality internal factors qualify the green belt of these three regions to have huge recreational potential. Additionally, the existence of the recreational green belt helps to meet the planning requirement of a 15-min walk to green space. Therefore, recreational transformation of the green belt in these three sections in order of priority will cope with the increased demand. 

## 4. Discussion

### 4.1. Influencing Factors of the Recreational Utilization of the Green Belt

In this study, the relationships between the internal and external attributes of the green belt and its recreational utilization were investigated. By comparing the influencing factors of the recreational utilization, it could be seen that the internal factors that had a great impact on the recreational potential of the green belt were the water area and the service area of 1500 m. The most influential accessibility factor was the number of subway stations, while the surrounding service facilities factors included the number of cultural facilities, the number of restaurants and the number of residential areas. The number of subway stations and the number of residential areas were negatively correlated, while the rest were positively correlated.

#### 4.1.1. Internal Factors

The results show that the scale of green spaces and water bodies had a positive impact on attracting residents to recreational activities. When increasing the area of green land and water bodies by 1%, the recreational potential of the green belt increases by 0.71%. The results confirm what previous studies described: that residents tend to use larger areas of green space [22,30,62], and that the existence of water bodies is attractive to residents regardless of the environment and accessibility [24,63]. 

The degree of beauty did not significantly affect the recreational utilization of the Shanghai green belt. Previous studies have found that residents are more willing to go to scenic urban green spaces [21]. The green belt, as a protective green space to restrain urban sprawl, did not take aesthetic beauty as an important concern in the initial planning and construction stage. Therefore, the degree of beauty of the green belt was generally low, which caused the lack of residents’ willingness to use it for recreation. At the same time, accessibility is an important factor affecting recreational utilization [64]. When infrastructure facilities such as road traffic and bus stations are not fully constructed and traffic accessibility is weak, residents are unwilling to go to the green belt for recreational activities, regardless of the beauty of the scenery [65]. It can be predicted that after the improvement of traffic accessibility and the completion of the plant landscape and supporting facilities, the degree of beauty may serve as a significant positive factor impacting the recreational utilization of the green belt.

It can be concluded that the size of green spaces and water bodies is a prerequisite for the recreational transformation of the green belt. The scale of green spaces and water bodies also guarantees the advantages of the green belt in terms of temperature regulation and atmospheric purification [66]. Therefore, in the process of recreational transformation, it is necessary to ensure the scale of green spaces and water bodies. On this basis, enriching the plant community, optimizing the plant seasonal view and cleaning the water body help increase the recreational utilization of the green belt while maintaining the ecological benefit, and achieve the purpose of developing and utilizing the compound utilization of the green belt. 

#### 4.1.2. Accessibility

With regard to accessibility, the number of subway stations has a negative impact on the recreational utilization of the green belt, which is different from the results in previous studies [26]. After dividing the green belt into pure forest belt and the park built along with the forest belt, regression analysis results indicate that the area of roads and the number of bus stops in the partial sections of the green belt (parks built on the green belt) are significantly positive factors affecting the recreational utilization of the green belt (Table 6). Previous quantitative studies showed that accessibility can increase the recreational use of green spaces [29]. However, qualitative analysis and research have shown that easily accessible green space does not necessarily lead to the use of green space [67]. Factors such as safety, night lighting and site flatness affect residents’ use of green space [68]. This explains why the road area and the number of bus stops are positively correlated with the recreational use of the green belt in the regression analysis results of the sections of the parks that have been built in this study. In the model of the pure green belts, due to the lack of night lighting facilities and safety facilities, the regression analysis results show that the two accessibility factors are not significantly correlated to the recreational use of the green belt. As of this moment, the main purpose of the Green Belt is still a forest belt with ecological protection. The subway station represents a time-consuming way for residents to travel, and is not the most convenient method of transportation. This explains why when the number of subway station increases by 1%, the recreational utilization will decrease by 0.665%. Although the research results did not explicitly conclude the positive effect of accessibility on the recreational use of green belts, it was found in related design practices that good traffic accessibility has become a prerequisite for renovation design [69,70]. Therefore, for the recreational transformation of the green belt, it is necessary to increase the convenience of transportation.

#### 4.1.3. Surrounding Service Facilities

The model suggests that if the number of cultural facilities and restaurants increases by 1%, this will lead to an increase in recreational utilization by 2.33%. The dining spots attract a massive flow of people, which is positively related to the further recreational use of the green belt [31,71]. The construction of cultural facilities helped to improve the economic value of the area, attract projects and investment, increase the cultural connotation and cultural heritage of the site, and stimulate the generation of public spaces. A green belt around a city can serve as a measure for residents to enter these cultural platforms, which is more effective for stimulating local economic development and spatial potential as well as producing a substantial effect on the recreational use of a green belt around a city [72]. In the regression analysis of built parks, cultural facilities and shopping spots were significantly negatively correlated with the recreational use of the green belt. One possible reason may be that the locations of built parks along the green belt are economically developed and densely populated areas, with relatively high-quality infrastructure service facilities and well-designed recreational spaces. Therefore, residents do not have to go to a green belt with relatively low accessibility for recreational activities. In addition, entertainment facilities were significantly negatively correlated as shown in Table 6 and Table 7. Entertainment facilities include cinemas, theaters, etc., which residents usually choose for certain entertainment purposes which typically last several hours. A previous study has shown that the leisure and recreation time of urban residents generally persists for 1–3 h [73], and after spending several hours in entertainment facilities people usually no longer spend time in the green belt for recreational activities. 

Residential green space has ecological effects, such as regulating the temperature and humidity inside the residential area and having strong noise reduction and dust retention [74,75]. After the introduction of the green space management policies in residential areas, the area of green space in residential areas has rapidly increased, providing a large amount of green space for the city [76]. The green space in the residential area connects the urban green space system, which provides basic ecosystem services for the city [77]. From the perspective of recreation, some studies believed that residential areas had particular importance and that surrounding residents had specific needs for daily recreational activities [31]. Therefore, a green belt located next to a residential area has the proper conditions for recreational development and utilization [78,79]. However, studies have also shown that if nearby green spaces are considered uncomfortable and unattractive, individuals will prefer not to participate in outdoor recreational activities [80]. The research results of this study on the Shanghai green belt tend to be close to the latter conclusion. The policy of the “Guidelines for Environmental Landscape Design of Residential Areas (Trial Draft)” enabled a better internal green space construction within the residential area after 2005, qualifying those residential green spaces for recreational use. Combined with the previous analysis of the degree of beauty and the traffic accessibility, the residential area was far from the green belt and the atheistic beauty was insufficient, so the residents tended to choose the more convenient internal green space of the residential area. This has been confirmed in some design investigations [69]. Designers found that there are also sites in the green belt with little utilization by surrounding residents due to poor accessibility, even after undergoing a recreational transformation. The existence of high-quality recreational green space in the residential areas and the lack of convenient transportation to the green bel might lead to the fact that the residential area in this study (Table 5 and Table 7) does not have a positive impact on recreational utilization. In response to the needs of residents in these residential areas, the green belts have to be transformed into recreational areas that are different from the recreational functions of the green spaces in the residential areas. By developing river entertainment projects through the design of waterfronts [81], the design of urban greenways provides broader sports and entertainment options [82]. These are recreational functions that cannot be included in residential green space, but can be realized in the green belt.

### 4.2. Recreational Transformation of the Ecological Green Belts

In the “Shanghai City Master Plan (2017–2035)”, the most important role of the Shanghai green belt is still to effectively suppress the spread of the surrounding areas of the central city and provide ecological benefits as an ecological corridor of the city. At the same time, the plan mentions the indicator requirements for a 15-min walking distance to reach a green space. In the context of the shortage of urban land, the green belt has great potential to make up for the lack of green space within a 15-min walk for surrounding residents. This means that some sections of the 98 km green belt need to increase parks, greenways and other spaces for recreation and leisure to meet the recreational needs of surrounding residents. The results of this study reinforce the belief that with urban development and urban renewal, some sections of a green belt have a high potential for recreational transformation, as shown in Figure 2 (a), (b) and (c). For sections with recreational potential, in addition to ecological service functions, attention should be paid to expanding the water body areas, optimizing the plant landscapes, and increasing the construction of internal service facilities. For traffic conditions, bus and subway stations should be optimized to increase traffic accessibility. For surrounding service facilities, it is necessary to continue to strengthen the construction of cultural facilities and the catering industry, and the construction of residential areas can be advocated to increase the potential service population of a green belt. For sections with a low recreational potential, the current ecological environment should continue to be maintained to better serve its ecological service functions for a city.

With the rapid development of urbanization, attention must be paid to the health and well-being of urban residents while controlling the scale of a city. Therefore, many countries pay attention to urban ecological protection green belts. They transform the traditional pure ecological protection green belts into partial recreational areas to deal with the shortage of urban green recreational space under the background of urban renewal in order to provide places for recreational activities, so that they can improve the health of urban residents. London has developed the “All London Green Grid” to provide opportunities for outdoor sports near urban areas [83]. The Green Belt Berlin puts the design of recreational spaces in its most important position [49]. Beijing has transformed a second green belt into a diverse recreational space [84]. The Pearl River Delta greenway provides public infrastructure and entertainment space for urban areas [50]. Shanghai has also incorporated the transformation into the planning direction of modern ecological protection green space. The results of this research will guide the specific location of the planned transformation and put forward new ideas for the recreational transformation of ecological protection green belts in high-density cities, and contribute to the promotion of social health.

## 5. Conclusions

Approaching 30 years since its establishment, the Shanghai green belt has achieved great success in microclimate adaptation, air pollution purification and other ecological effects [85]. With the development of the city of Shanghai and the increasing demand for recreation from surrounding citizens, it is urgent to explore the comprehensive function of the Shanghai green belt and transform some pure ecological forests for recreational utilization. In this study, social media data was used to analyze the factors affecting recreational utilization along the Shanghai green belt. Multiple regression models were used to analyze the relationship between the recreational utilization of the green belt and the influencing factors.

The regression model results showed that the number of cultural facilities, the areas of the water bodies, the number of restaurants and the total external area had a significant positive influence on the recreational utilization of the Green Belt. However, the number of residential areas was negatively correlated with the number of subway stations. However, after the verification of the segmentation models and the analysis of the literature, we found that the number of residential areas and the accessibility of traffic are important factors that affect the recreational utilization of the Green Belt. It is recommended that the recreational transformation planning of a green belt should consider the characteristics of the green belt and the completeness of the surrounding service facilities, as described in the paragraphs with recreational potential in this study, for example, Figure 2 (a), (b) and (c). On the purpose of ensuring the ecological effects already possessed by the green belt, the plant community structure should be optimized, the seasons should be enriched and recreational transformation should be carried out according to the needs of residents in the surrounding residential areas. While in some areas with a small amount of green space, an imperfect road traffic system and few potential populations, it is still better to maintain the primary function of ecological protection of the green belt, playing the role of adjusting temperature and humidity (alleviating the urban heat island effect) as well as purifying the atmospheric environment to protect the natural environment of the city.

The result of this study provides a guideline for the sustainable development of urban ecological protection green belts for urban renewal. It contributes to the research on the compound functions of green belts and new green belt planning policies. The study proposes planners incorporate the recreational transformation of urban ecological protection green belts into planning considerations. The improvement of the recreational utilization of the green belt will put forward requirements for the penetration of roads, the construction of residential areas and the layout of cultural facilities in addition to other public service facilities outside the green belt, and will guide planners to make reasonable plans for the city. At the same time, the green belt after the recreational transformation will provide urban residents with practically accessible urban green spaces and balance the physical and psychological negative effects of urbanization on residents, contributing to the promotion of social health. This study proved that it is feasible to analyze the recreational utilization of a green belt through social media data, and it has important guiding significance for the planning of a green belt, especially the recreational transformation with the background of urban regeneration. However, there are still some limitations in this study. First, although the Shanghai Statistical Yearbook showed that the basic data within the scope of the study does not vary too much, it is not possible to separate the data by time ranging over 8 years, due to the lack of that information from open source websites, and therefore the analysis results would be slightly affected. Secondly, social media data has the advantages of a large amount of data and a large time span. However, there will be restrictions on the ages of the crowd members. Big data does not mean complete data. The Weibo sign-in data that was used was actually limited by the type of demographic. A survey showed that the recreational population in the green belt was mainly made up of middle-aged and elderly people. Among this population, the elderly people did not use social media frequently. When using big data, there will be a lack of relevant data, which will have a certain impact on the final results. In future studies, social media data can be combined with questionnaire surveys to make up for the lack of information on the use of green belts by users of all ages that cannot be proven by social media data. Finally, the different impacts of several influencers in the results of the three models in this study come from the difference of development degree and geographical space along the green belt. These differences will be further studied in future research.

## Figures and Tables

**Table 1:** Social media data information.

Data	Time	Quantity of Records	Fields	Use
Sina Weibo check-in data	December 2011–July 2020	3,019,644	User ID, number of points, longitude, latitude and time	To identify the destinations of users

**Table 2:** Basic data information related to the green belt.

Data	Time	Quantity	Use
Road network	2019	12,273	To determine the traffic convenience of the green belt
Bus station	2020	7284	
Subway station	2020	37	
POI data	2020	24,088	To determine the commercial prosperity of the green belt

**Table 3:** Information on the potential factors affecting the recreational utilization of the green belt.

Type	Factors	Description	Measurement
Internal factors	The size of the green area	The land coverage area of the green area	hm^2^
	The size of the water body	The land coverage area of the water area	hm^2^
	The size of the 1500-m area	The land coverage area of the 1500-m buffer zone of the green belt	hm^2^
	The degree of beauty	Evaluation score for green belt scenery	Grade
Accessibility	Road area	The land coverage area of the road network	hm^2^
	Bus station	The number of bus stations	Count
	Subway station	The number of subway stations	Count
Surrounding service facilities	Residential areas	The number of residential areas	Count
	Shopping spots	The number of shopping spots, such as shopping malls, convenience stores, hypermarkets, supermarkets, commercial streets and specialty stores	Count
	Restaurants	The number of restaurants, such as Chinese and foreign restaurants, fast food restaurants, coffee shops, teahouses and pastry shops	Count
	Cultural facilities	The number of cultural facilities, such as museums, exhibition halls, art galleries, libraries, science and technology museums, cultural palaces, archives and schools	Count
	Tourist spots	The number of tourist spots, such as zoos, botanical gardens and scenic spots	Count
	Entertainment facilities	The number of entertainment facilities, such as sports venues, entertainment places, theaters and vacation resorts	Count

**Table 4:** Summary of statistics of variables used.

Variable Category	Variable Name	Minimum	Maximum	Mean	Standard Deviation
Internal factors	The green area (hm^2^)	1.34	485.26	204.24	108.05
	The water area (hm^2^)	0.00	39.61	6.36	10.24
	The 1500-m buffer zone (hm^2^)	1307.41	2,404,560.00	44,985.60	318,165.34
	The degree of beauty (grade)	2.00	8.00	4.60	1.33
Accessibility	Road area (hm^2^)	120.11	800.88	286.75	138.95
	Bus station (count)	0.00	5.00	1.36	1.32
	Subway station (count)	28.00	815.00	238.46	166.84
Surrounding service facilities	Residential areas (count)	0.00	139.00	28.32	25.45
	Shopping spots (count)	0.00	32.00	7.98	6.65
	Restaurants (count)	0.00	182.00	35.61	34.64
	Cultural facilities (count)	2.00	23.00	8.00	4.97
	Tourist spots (count)	0.00	8.00	2.64	2.22
	Entertainment facilities (count)	0.00	41.00	8.09	7.96

**Table 5:** Results of the multivariate regression model.

Model Variable		Coeff.	PCSE
Intercept		−1.944	2.355
	ln (the size of the green area)	0.334	0.199
	ln (the size of the water body)	0.417	0.088 ***
	ln (the size of the 1500-m area)	0.293	0.096 ***
	ln (the degree of beauty)	0.487	0.466
	ln (road area)	0.411	0.527
	ln (bus station)	−0.873	0.225
	ln (subway station)	−0.665	0.326 **
	ln (residential areas)	−0.668	0.199 ***
	ln (shopping spots)	0.263	0.229
	ln (restaurants)	1.124	0.205 ***
	ln (cultural facilities)	1.204	0.305 ***
	ln (tourist spots)	−0.235	0.18
	ln (entertainment facilities)	−0.263	0.176
Overall model			
sample size (n)	192		
Adjusted R^2^	0.533		
F-test	14.628 ***		

*NOTES:* Coeff. = coefficient; PCSE = panel-corrected standard errors. *** = significant at 99% (*p* ≤ 0.01); ** = significant at 95% (*p* ≤ 0.05); * = significant at 90% (*p* ≤ 0.10).

**Table 6:** Regression results for the parks and pure green belts.

Model Variable		Parks Built on Green Belts		Pure Green Belts	
		Coeff.	PCSE	Coeff.	PCSE
Intercept		80.088	13.869 ***	1.578	2.678
	ln (the size of the green area)	8.706	0.787 ***	0.069	0.223
	ln (the size of the water body)	7.311	1.002 ***	0.136	0.135
	ln (the size of the 1500-m area)	5.933	4.710 ***	0.309	0.094 ***
	ln (the degree of beauty)	0.001	0.395	0.132	0.558
	ln (road area)	21.277	2.677 ***	−0.047	0.653
	ln (bus station)	11.587	1.837 ***	−0.362	0.367
	ln (subway station)	−2.094	0.329 ***	−0.987	0.334 ***
	ln (residential areas)	−2.82	0.398 ***	−1.344	0.304 ***
	ln (shopping spots)	−26.406	3.781 ***	0.702	0.260 ***
	ln (restaurants)	14.579	1.953 ***	1.134	0.239 ***
	ln (cultural facilities)	−11.744	1.908 ***	1.234	0.424 ***
	ln (tourist spots)	4.452	0.779 ***	0.205	0.219
	ln (entertainment facilities)	−1.624	0.354 ***	−0.294	0.223
Overall model					
sample size (n)		51		139	
Adjusted R^2^		0.928		0.445	
F-test		42.278 ***		7.964 ***	

*NOTES:* Coeff. = coefficient; PCSE = panel-corrected standard errors. *** = significant at 99% (*p* ≤ 0.01); ** = significant at 95% (*p* ≤ 0.05); * = significant at 90% (*p* ≤ 0.10).

**Table 7:** Regression results for the residential areas built before 2005 and after 2005.

Model Variable		Residential Areas Built before 2005		Residential Areas Built after 2005	
		Coeff.	PCSE	Coeff.	PCSE
Intercept		−3.409	7.862	−2.787	3.757
	ln (the size of the green area)	−0.596	0.254 *	1.180	0.308 ***
	ln (the size of the water body)	1.011	0.146 ***	0.057	0.179
	ln (the size of the 1500-m area)	1.346	1.362	0.260	0.106 **
	ln (the degree of beauty)	−0.001	0.682	1.464	0.651 **
	ln (road area)	0.653	0.943	−0.638	0.906
	ln (bus station)	−1.941	0.576 ***	−0.381	0.445
	ln (subway station)	−0.758	0.302 **	−0.777	0.405 *
	ln (residential areas)	−0.454	0.507	−0.681	0.291 **
	ln (shopping spots)	0.055	0.543	0.553	0.296 *
	ln (restaurants)	2.721	0.479 ***	0.880	0.286 ***
	ln (cultural facilities)	2.011	0.445 ***	1.068	0.555 *
	ln (tourist spots)	0.003	0.314	−0.532	0.294 *
	ln (entertainment facilities)	−2.300	0.439 ***	−0.083	0.217
Overall model					
sample size (n)		83		103	
Adjusted R^2^		0.705		0.522	
F-test		13.403 ***		8.022 ***	

*NOTES:* Coeff. = coefficient; PCSE = panel-corrected standard errors. *** = significant at 99% (*p* ≤ 0.01); ** = significant at 95% (*p* ≤ 0.05); * = significant at 90% (*p* ≤ 0.10).

## Data Availability

The data presented in this study are available on request from the corresponding author. The data are not publicly available due to privacy.
